# Genetic Contribution of MHC Class II Genes in Susceptibility to West Nile Virus Infection

**DOI:** 10.1371/journal.pone.0165952

**Published:** 2016-11-03

**Authors:** Constantina A. Sarri, Maria Markantoni, Costas Stamatis, Anna Papa, Athanasios Tsakris, Danai Pervanidou, Agoritsa Baka, Constantina Politis, Charalambos Billinis, Christos Hadjichristodoulou, Zissis Mamuris, MALWEST project

**Affiliations:** 1 Laboratory of Genetics, Comparative and Evolutionary Biology, Department of Biochemistry and Biotechnology, University of Thessaly, Larissa, Greece; 2 Arboviruses Reference Laboratory, 1st Microbiological Laboratory, Aristotle University of Thessaloniki, Thessaloniki, Greece; 3 Laboratory of Microbiology, Medical School, National and Kapodistrian University of Athens, Athens, Greece; 4 Hellenic Centre for Disease Control and Prevention (HCDCP), Attika, Greece; 5 Hellenic coordinating Hemovigilance Center of HCDCP, Attika, Greece; 6 Laboratory of Microbiology and Parasitology, Faculty of Veterinary Medicine, University of Thessaly, Karditsa, Greece; 7 Laboratory of Hygiene and Epidemiology, Faculty of Medicine, University of Thessaly, Larissa, Greece; Universidade Nova de Lisboa Instituto de Higiene e Medicina Tropical, PORTUGAL

## Abstract

WNV is a zoonotic neurotropic flavivirus that has recently emerged globally as a significant cause of viral encephalitis. The last five years, 624 incidents of WNV infection have been reported in Greece. The risk for severe WNV disease increases among immunosuppressed individuals implying thus the contribution of the MHC locus to the control of WNV infection. In order to investigate a possible association of MHC class II genes, especially *HLA-DPA1*, *HLA-DQA1*, *HLA-DRB1*, we examined 105 WNV patients, including 68 cases with neuroinvasive disease and 37 cases with mild clinical phenotype, collected during the period from 2010 to2013, and 100 control individuals selected form the Greek population. Typing was performed for exon 2 for all three genes. DQA1*01:01 was considered to be "protective" against WNV infection (25.4% vs 40.1%, *P* = 0.004) while DQA1*01:02 was associated with increased susceptibility (48.0% vs 32.1%, *P* = 0.003). Protection against neuroinvasion was associated with the presence of DRB1*11:02 (4.99% vs 0.0%, *P* = 0.018). DRB1*16:02 was also absent from the control cohort (*P* = 0.016). Three additional population control groups were used in order to validate our results. No statistically significant association with the disease was found for *HLA-DPA* alleles. The results of the present study provide some evidence that MHC class II is involved in the response to WNV infection, outlining infection "susceptibility" and "CNS-high-risk" candidates. Furthermore, three new alleles were identified while the frequency of all alleles in the study was compared with worldwide data. The characterization of the MHC locus could help to estimate the risk for severe WNV cases in a country.

## Introduction

West Nile Virus (WNV) is a zoonotic neurotropic arbovirus of the Flaviviridae family that has recently emerged globally as a significant cause of viral encephalitis [1]. During the last seven years, disease outbreaks with neuroinvasive human cases have been reported in several European countries [2]. In Greece, 624 incidents of WNV infection have been reported since 2010. The majority (446 patients) developed neuroinvasive symptoms, while 79 fatalities were recorded [3]. The high proportion of neuroinvasive cases, which is not a WNV-disease characteristic, reflects the majority of unreported subclinical or asymptomatic infections.

The majority of WNV infections are asymptomatic while approximately 1/5 of the infected individuals develop WNV fever. An even smaller proportion (<1%) develop neuroinvasive disease which includes meningitis, encephalitis, and acute flaccid paralysis (AFP)/poliomyelitis [4]. The risk for developing a more severe form of WNV disease increases among immunosuppressed individuals and organ transplant recipients [5], the elderly [6, 7] and men [8]. Indeed, immunosuppression due to defective CD4+ and CD8+ T-cell response was shown to contribute to WNV infection of the Central Nervous System (CNS) and increased mortality rates [9–11]. The major histocompatibility complex (MHC) class I polymorphism has also been associated with the outcome of WNV infection; in an American cohort study, HLA-B*40 and C*03 were attributed a protective role contrary to HLA-A*68 and C*08 that were associated with the development of neurological symptoms [12]. The contribution of HLA-A locus to the development of immune responses that control WNV infection was also recently supported by mass spectroscopy data [13].

Although presentation of viral antigens relies classically on MHC class I molecules, sufficient T-cell CD4+ engagement is an important predictor of outcome for several viral infectious diseases, including hepatitis A and B, and influenza [14]. T-cell CD4+ receptors are the direct interactors of MHC class II molecules and MHC class II genes have been associated with the outcome of many viral infections such as those caused by HBV, HCV, HIV, EBV and dengue viruses [14–21] including the severity of WNV disease [12]. These data suggest a pivotal role of MHC class II system in viral disease fight.

MHC class II molecules consist of two chains, α and β. Both of them contribute to the formation of the peptide-binding region (PBR). DPA, DQA and DRA genes code for α chain, while DPB, DQB and DRB genes code for β chain. With the exception of DRA which is essentially monomorphic, the MHC class II locus is highly polymorphic. The high polymorphism that is observed is mainly located in exon 2 of these molecules that codes for PBR. The array of PBR variation determines the range of recognizable pathogen-derived antigens that are subsequently presented to T-cell receptors to initiate the immune response [22].

In the present study, the polymorphism of exon 2 of *HLA-DPA*1, *HLA-DQA*1 and *HLA-DRB*1 genes was examined in both control and WNV cases among the Greek population. The present study offers valuable insight regarding the possible influence of the MHC class II background in the infection and outcome of the WNV disease. This information is essential as the basis for developing diagnostic tools of high predictive power and for matching vaccine antigens to the immune background of human populations.

## Materials and Methods

A total of 105 confirmed WNV cases were identified during a 4-year period (2010–2013) from different regions in Greece including Central Greece, Attica, Central Macedonia, Eastern Macedonia and Thrace. Sixty eight persons were reported as having encephalitis (including meningoencephalitis), meningitis or acute flaccid paralysis and were classified as having West Nile Neuroinvasive Disease (WNND). WNND classification was based on the treating physicians’ clinical assessment and laboratory data (detection of WNV nucleic acid and/or WNV-specific antibody response in cerebrospinal fluid (CSF) and/or imaging findings). Thirty seven persons infected with WNV developed a mild disease referred to as West Nile Fever (WNF). All samples and data were derived from two diagnostic laboratories designated by the Hellenic Center for Disease Control and Prevention (HCDCP—KEELPNO); the Reference Laboratory for Arboviruses, Aristotelian University, Thessaloniki and the Department of Microbiology, School of Medicine, University of Athens. Due to the small case-sample, no filtration was used and all cases were included in the study.

Moreover 100 individuals were used as control group. Control samples were derived from prefecture of Thessaly. According to a recent study, the general Greek population is genetically homogenous, except for some special minorities that represent genetic isolates [23]. Neither of our samples (WNV cases and healthy control individuals) included individuals from these isolates. In this study, a case control has been defined as a person for whom negative results were obtained in the assessment of the following laboratory criteria: isolation of WNV from blood or CSF, detection of WNV nucleic acid in blood or CSF, WNV-specific antibody response (IgM) in CSF and WNV IgM high titer, detection of WNV IgG, and confirmation by neutralisation. Based on the lack of WNV IgG and IgM detection, we can only assume that these individuals have not been exposed to WNV. This control group fails to match by WNV exposure with the cases. However, WNV disease is mostly asymptomatic, thus making the finding of the most fitted control group a matter of luck, since these individuals can only be tracked incidentally. So, as a second most fitted control group, we chose to compare allelic frequencies between cases and non-exposed groups. The controls were matched by age (five year interval) and sex with the cases. The case group consisted of both WNND and WNF. The contrast between WNND and WNF symptoms inevitably raise the question whether the MHC polymorphism has a role in neuroinvasion. Thus, WNV cases cohort and both its subgroups were compared to healthy control individuals.

For this study, we selected genes encoding for antigen-binding proteins of the MHC class II cluster, with low (*HLA-DPA1*), medium (*HLA-DQA1*) and high (*HLA-DRB1*) level of polymorphism. *HLA-DRB1* is the most polymorphic class II gene. *HLA-DQB1*, *HLA-DPB1* and *HLA-DQA1* genes show lower variability. Due to the small sample available and the high MHC polymorphism, *HLA-DQA1* was chosen as a “safer” gene to explore the potential genetic contribution of the MHC class II alleles to WNV.

DNA isolation was performed using PureLink Genomic DNA Mini Kit (Invitrogen) according to the manufacturer’s instructions. Amplification of exon 2 of *HLA-DPA1*, *HLA-DQA1* and *HLA-DRB1* was performed using the appropriate primers: *HLA-DPA1* exon 2 [Fw: CTCTAGCTTTGACCACTTGC and Rv: CTTCCAGTTGGGCTACAGAG (Ta = 59°C)]; *HLA-DQA1* exon 2 [Fw: CGCCTTCCTGCTTGTCATC and Rv: AGGCAGAATGGTGGACGC (Ta = 56°C)]; *HLA-DRB1* exon 2 [Fw: CGTGTCTTCTCAGGAGGC and Rv: CTCAGATTCCCAGCTCACG (Ta = 57°C)]. PCR reactions (50μl) contained 200ng DNA, 1X Taq buffer, 2 mM MgCl_2_, 0.2mM of each dNTP, 50 pmol of each primer and 1 U Taq DNA polymerase (KAPA Biosystems). The cycling conditions consisted of an initial denaturation at 95°C for 4 min followed by 35 cycles of denaturation at 95°C for 40 s, annealing at Ta°C for 40 s and extension at 72°C for 30–40 s, with a final extension at 72°C for 10 min. The primers used were designed to amplify exon 2 of the three genes and produce a fragment <400bp, in order to perform Single-Strand Conformation Polymorphism (SSCP) analysis.

Exon 2 of *HLA-DPA1* and *HLA-DQA1* were screened using the SSCP method using silver staining to visualize DNA bands. Samples with different profiles were sequenced. Concerning *HLA-DRB1*, all samples were sequenced and the PCR products were purified using NucleoSpin Gel and PCR Clean-up kit (Macherey-Nagel) according to the manufacturer’s instructions. Sequencing was performed by Macrogen Inc. To compare the allele sequences identified in this study with those already deposited in public databanks, we used the EBI BLAST engine.

The association of each allele with WNV infection outcome was examined with pairwise comparison using the z test (XLSTAT 2015.1.01) and the Bonferroni method to adjust p-values for multiple comparisons. Comparison of homozygosity and heterozygosity frequencies for each gene was held with Fisher’s exact two-tailed test. A p-value less than 0.05 was considered statistically significant.

Alleles showing statistically significant different distribution between cases and our control group or an association trend were further checked. For this purpose, we used four population control groups [24]. All of the population groups that were chosen met the following criteria: 1) they were collected before 2010, when WNV first appeared in Greece [3] and 2) included over 100 individuals; thus, we ensured that a large sample would be available for confirmation/contradiction of the obtained results, overcoming the high diversity of the MHC that might not be represented in a small population sample. The designation of population control groups that were collected prior to 2010, was made in order for them to belong in the non-exposure group, as our controls. The populations used for DQA analysis were Greece pop2 (n = 120), Greece pop3 (n = 246) and Greece pop 5 (n = 500). For DRB allele analysis the control groups used were Greece Crete (n = 135), Greece pop 2 (n = 120) and Greece pop 3 (n = 246) [24]. These populations included individuals that originated from all over Greece, matching the source of the cases.

### Ethics Statement

All participants were informed by the investigators about the study and they gave their written consent in order to participate. Both the study and the consent procedure were approved by the ethics committee of the Medical Faculty of the University of Thessaly.

## Results

Five alleles in *HLA-DPA*1, seven alleles in *HLA-DQA*1 and 24 alleles in *HLA-DRB*1 were identified (Table 1).

**Table 1:** The official nomenclature of the identified alleles and their frequency in European populations together with the respective allelic frequencies found in this study (column Greece) [24].

	Official name	Allele frequency					
		Europe	Greece	North Africa	Sub-Saharan Africa	Western Asia	South Asia
**1**	DPA1*01:03	0.767–0.865	0.844	no info	0.095–0.425	no info	0.593
**2**	DPA1*02:01	0.025–0.188	0.104	no info	0.0–0.558	no info	0.212
**3**	DPA1*02:02	0.004–0.14	0.043	no info	0.0–0.241	no info	0.068–0.093
**4**	DPA1*01:05	0.017	0.005	no info	no info	no info	no info
**5**	DPA1*03:01	0.0–0.005	0.005	no info	0.082–0.266	no info	0.0
**1**	DQA1*01:02	0.0–0.337	0.404	0.121–0.289	0.182–0.5	0.069–0.287	0.029–0.156
**2**	DQA1*01:01	0.0–0.268	0.324	0.042–0.168	0.051–0.197	0.044–0.425	0.032–0.188
**3**	DQA1*01:03	0.0181–0.239	0.100	0.015–0.044	0.012–0.08	0.008–0.163	0.081–0.271
**4**	DQA1*03:01	0.005–0.2037	0.112	0.103–0.142	0.004–0.07	0.06–0.241	0.042–0.241
**5**	DQA1*02:01	0.007–0.313	0.044	0.123–0.223	0.029–0.12	0.035–0.269	0.069–0.285
**6**	DQA1*05:01	0.0–0.518	0.012	0.126–0.286	0.117–0.401	0.086–0.395	0.024–0.215
**7**	no reference	-	0.003	-	-	-	-
**1**	DRB1*11:04	0.006–0.196	0.255	0.0–0.044	0.0–0.037	0.004–0.237	0.0–0.019
**2**	DRB1*11:01	0.009–0.205	0.103	0.0–0.137	0.006–0.144	0.022–0.302	0.045–0.12
**3**	DRB1*11:18	0.0001	0.009	0.0	0.0	0.019	no info
**4**	DRB1*14:01	0.004–0.077	0.054	0.0–0.02	0.0–0.036	0.0–0.08	0.009–0.074
**5**	DRB1*13:01	0.01–0.217	0.063	0.01–0.21	0.016–0.241	0.008–0.154	0.011–0.138
**6**	DRB1*03:01	0.01–0.557	0.083	0.0–0.202	0.022–0.181	0.017–0.29	0.016–0.54
**7**	DRB1*08:30	0.000	0.003	no info	no info	no info	no info
**8**	^*^DRB1*16:02	0.003–0.018	0.011	no info	0.008–0.031	0.003	0.009
**9**	DRB1*16:01	0.002–0.155	0.111	0.0–0.03	0.006–0.016	0.0–0.061	0.0–0.019
**10**	DRB1*15:01	0.015–0.3	0.097	0.0–0.134	0.0–0.112	0.005–0.21	0.004–0.194
**11**	DRB1*15:02	0.0–0.106	0.037	0.0–0.066	0.0–0.024	0.004–0.085	0.008–0.141
**12**	DRB1*13:05	0.0–0.022	0.014	0.0–0.005	0.0	0.0–0.031	0.0
**13**	DRB1*08:01	0.0008–0.198	0.006	0.0–0.036	0.0–0.015	0.0–0.044	0.005–0.012
**14**	DRB1*13:02	0.006–0.078	0.029	0.024–0.173	0.0–0.159	0.003–0.196	0.0–0.059
**15**	DRB1*08:04	0.0–0.025	0.020	0.0–0.12	0.0–0.075	0.0–0.051	0.0
**16**	DRB1*11:02	0.0–0.027	0.009	0.0–0.082	0.0–0.144	0.0–0.025	0.0–0.013
**17**	DRB1*14:04	0.0–0.160	0.011	0.0–0.01	0.0–0.005	0.0–0.032	0.0–0.13
**18**	DRB1*10:01	0.0–0.046	0.023	0.009–0.073	0.0–0.189	0.005–0.153	0.011–0.147
**19**	DRB1*12:01	0.0–0.052	0.026	0.0–0.035	0.008–0.052	0.0–0.042	0.003–0.102
**20**	DRB1*13:03	0.0–0.083	0.011	0.005–0.057	0.0–0.081	0.0–0.076	0.0–0.157
**21**	DRB1*11:03	0.0–0.041	0.009	0.0–0.01	0.0	0.0–0.205	0.003–0.016
**22**	^‡^DRB1*11:15 (13:62)	0.0–0.0012	0.003	no info	0.0	no info	no info
**23**	DRB1*11:192	-	0.006	-	-	-	-
**24**	^‡^DRB1*08	0.0–0.223	0.006	0.014–0.192	0.006–0.18	0.002–0.03	0.0–0.134

‡Higher resolution was not feasible to achieve for these alleles.

Three novel alleles out of 36 in total were identified during this study, not previously reported at a global scale. DPA5 and DRB23 alleles have been officially named DPA1*03:01 (1991) and DRB1*11:192 (2015) respectively [25, 26]. Concerning the DQA7 allele, the inability to sequence more *HLA-DQA1* exons, terminated the process for an official nomenclature assignment.

### *HLA-DPA*1

Of the five alleles identified in *HLA-DPA*1, DPA1*01:03 allele was the most abundant (>83% in all groups). DPA1*01:05 and DPA1*03:01 alleles were observed in low frequencies (<2%). The DPA1*01:05 allele was present exclusively in the WNND group (Table 2). Due to lack of statistical significance for the comparisons between our cases and control groups, no further analysis was performed.

**Table 2:** Pair-wise analysis of the MHC class II frequencies in controls, total patients and WNND/WNF* subpopulations. P values in bold indicate significance.

Allele	Controls	Cases	Cases vs Controls	Cases		WNND vs Controls	WNND vs WNF
				WNND	WNF		
*Allele %*							
**DPA**	**n = 195**	**n = 203**	**p-value**	**n = 136**	**n = 67**	**p-value**	**p-value**
DPA1*01:03	85.1	83.7	0.937	83.1	85.1	0.623	0.716
DPA1*02:01	10.3	10.4	0.973	10.2	10.5	0.977	0.947
DPA1*02:02	4.1	4.4	0.882	4.4	4.5	0.894	0.974
DPA1*01:05	0.0	1.0	0.162	1.5	0.0	0.726	0.314
DPA1*03:01	0,5	0.5	1.000	0.7	0.0	0.086	0.492
**DQA**	**n = 162**	**n = 177**	** p-value**	**n = 115**	**n = 62**	**p-value**	**p-value**
DQA1*01:02	32.1	48.0	**0.003**	52.2	40.3	**0.001**	0.131
DQA1*01:01	40.1	25.4	**0.004**	28.7	19.4	0.051	0.175
DQA1*01:03	11.1	9.0	0.520	6.1	14.5	0.152	0.063
DQA1*03:01	11.1	11.3	0.953	8.7	16.1	0.514	0.138
DQA1*02:01	4.9	4.0	0.687	2.6	6.5	0.333	0.205
DQA1*05:01	0.0	2.3	0.052	1.7	3.2	0.096	0.519
**No reference**	0.6	0.0	0.302	0.0	0.0	0.405	
**DRB**	**n = 168**	**n = 179**	** p-value**	**n = 115**	**n = 64**	**p-value**	**p-value**
DRB1*11:04	28.6	22.9	0.224	22.2	23.4	0.228	0.854
DRB1*11:01	12.5	8.4	0.198	9.4	6.3	0.415	0.470
DRB1*11:18	1.2	0.6	0.551	0.0	1.6	0.234	0.170
DRB1*14:01	7.7	3.4	0.070	5.1	0.0	0.386	0.066
DRB1*13:01	8.3	3.4	0.133	4.3	4.7	0.183	0.901
DRB1*03:01	8.3	8.4	1.000	7.7	9.4	0.855	0.692
DRB1*08:30	0.0	0.6	0.315	0.0	1.6	-	0.170
DRB1*16:02	0.0	2.2	0.053	3.4	0.0	**0.016**	0.136
DRB1*16:01	8.3	13.9	0.098	11.1	18.8	0.429	0.153
DRB1*15:01	10.7	8.9	0.549	10.3	6.3	0.914	0.366
DRB1*15:02	2.4	5.0	0.201	4.3	6.3	0.369	0.555
DRB1*13:05	1.2	1.7	0.697	1.7	1.6	0.724	0.960
DRB1*08:01	1.2	0.0	0.139	0.0	0.0	0.234	-
DRB1*13:02	3.6	2.2	0.434	1.7	3.1	0.341	0.539
DRB1*08:04	0.6	3.4	0.072	4.3	1.6	0.033**	0.334
DRB1*11:02	0.0	1.7	0.090	0.0	4.7	-	**0.018**
DRB1*14:04	0.0	2.2	0.053	2.6	1.6	0.036**	0.664
DRB1*10:01	1.2	3.4	0.251	1.7	6.3	0.748	0.099
DRB1*12:01	2.4	2.8	0.815	4.3	0.0	0.369	0.092
DRB1*13:03	0.6	1.7	0.340	2.6	0.0	0.161	0.193
DRB1*11:03	1.2	0.6	0.551	0.0	1.6	0.234	0.170
DRB1*11:15 (13:62)	0.0	0.6	0.315	0.9	0.0	0.218	0.447
DRB1*11:192	0.0	1.1	0.173	0.9	1.6	0.218	0.672
DRB1*08	0.0	1.1	0.173	1.7	0.0	0.090	0.294

*WNND (West Nile Neuroinvasive Disease), WNF (West Nile Fever).

**No significant using the Bonferroni method.

### *HLA-DQA*1

In *HLA-DQA*1, seven alleles were identified. The DQA1*01:02 allele was observed at a significantly higher frequency in cases than in control subjects (*P* = 0.003) and in WNND patients in particular (Table 2). On the contrary, the DQA1*01:01 allele was identified at a significantly lower frequency in cases than in control subjects (*P* = 0.004). The DQA1*01:03, DQA1*03:01 and DQA1*02:01 allelic frequencies were over two times higher in WNF compared with WNND patients, respectively (Table 2). The DQA1*05:01 allele was absent from the control group whereas the DQA7 allele was found only in this group with a frequency of 0.6% (Table 2).

DQA1*01:01, DQA1*01:02 and DQA1*05:01 showed association or an association trend with WNV infection. Thus, the cases’ frequency was further compared with population control groups. DQA1*01:01 and DQA1*01:02 were found in significantly higher frequencies in our cases compared with all of these control groups (*P* < 0.0001, data not shown). Although the results for DQA1*01:02 agreed with the comparison with our control samples, for DQA1*01:01 they were reversed. Finally, for the DQA1*05:01 allele, cases showed a statistically significant lower frequency in comparison with two population control groups after Bonferroni correction (*vs* Greece pop 2 and Greece pop 3 *P*>0.0001, data not shown).

### *HLA-DRB*1

Twenty-four alleles were identified in *HLA-DRB*1. DRB1*08:01 appeared to be protective against WND and was present only in control subjects (Table 2). Seven alleles (DRB1*16:02, DRB1*11:02, DRB1*14:04, DRB1*11:192, DRB1*08:30, DRB1*08, DRB1*11:15) were unique to the cases group (Table 2). Three alleles (DRB1*16:02 (*P* = 0.016), DRB1*08, DRB1*11:15) were strongly associated in neuroinvasion as they were present only in WNND individuals (Table 2). Comparison of the allelic distribution between WNND and control group also revealed DRB1*08:04 and DRB1*14:04 alleles as associated with susceptibility to WNV (*P* = 0.033 and *P* = 0.036 respectively; not significant after the Bonferroni correction)]. On the contrary, alleles DRB1*11:18, DRB1*11:02 (*P* = 0.018), DRB1*11:03, DRB1*08:30 were found to be associated with protection against neuroinvasion, as none of the cases bearing them developed WNND (Table 2).

DRB1*08:04 and DRB1*14:04 alleles’ association with WNV infection was further verified in Greece pop 3 (*P* = 0.002* and *P* = 0.027 respectively, data not shown; *significant after the Bonferroni correction). Additionally, the association trend of DRB1*14:01 and DRB1*16:01 was confirmed when using larger sample (vs Greece pop 3, P = 0.033 and P = 0.015* respectively, data not shown; *significant after the Bonferroni correction).

### MHC class II genotype of deceased WNV cases

During the study, seven out of 105 patients deceased. All deaths were attributed to WNV disease. All of the cases belonged to the immunocompromised group, were over 60 years old with underlying diseases such as heart disease and diabetes (data not shown). No correlation with sex was observed (four male and three female cases). The number of the deceased cases was not sufficient to provide any correlation with sex although neurological symptoms were indeed associated with males (45 male and 23 female WNND cases, *P* = 0.008; data not shown). Although these findings are in agreement with the literature, they should be taken into account with caution because of the small cohort studied. Genotyping was not successful for all cases in the three loci (failure of amplification). In *HLA-DPA1*, DPA1*01:03 allele was found in all of the cases (12/14 alleles) and DPA1*02:01 allele in two cases in heterozygosity. Concerning *HLA-DQA1*, 80% of the WNV cases were homozygous for DQA1*01:02 allele (four cases) while DQA1*03:01 allele was also found (one case). Exon 2 of *HLA-DRB1* was amplified in four cases and no alleles showed any trend for association with severe disease.

### Comparison of the homozygosity state between controls and WNV cases groups

The proportion of homozygotes between controls, WNV cases and their subgroups was similar, concerning *HLA-DPA*1. In *HLA-DQA*1, a statistically significant difference was observed when comparing WNV cases vs controls and WNF vs controls (*P =* 0.0142 and *P =* 0.0338 respectively). Over 90% of the WNF subjects and almost 90% of the WNV cases were homozygous. In contrast, homozygosity in controls appeared in 75% of the group. In *HLA-DRB*1, the two WNV cases subgroups showed similar percentage of homozygosity (57.6%–59.4%). However, when WNV cases were compared with the control group, heterozygosity was associated with disease (*P* = 0.0382, WNND vs Controls *P* = 0.0484). Finally, in all three genes homozygosity was not associated with neuroinvasion, based on the different distribution of alleles between WNND and WNF groups (*HLA-DPA*1 *P* = 0.8193, *HLA-DQA*1 *P* = 0.4869 and *HLA-DRB*1 *P* = 1.000) (Table 3).

**Table 3:** Pair-wise analysis of homozygous/heterozygous state in controls, total WNV cases and WNND/WNF* subpopulations. *P* values in bold indicate significance^.^

Gene	Controls	Cases	Cases vs Controls	Cases		WNND vs Controls	WNND vs WNF
				WNND	WNF		
**DPA**	**n = 99**	**n = 102**	p-value**	**n = 68**	**n = 34**	p-value**	p-value**
**Homo**	71 (71.7%)	73 (71.6%)	1.000	48 (70.6%)	25 (73.5%)	1.000	0.8193
Hetero	**28 (28.3%)**	**29 (28.4%)**		**20 (29.4%)**	**9 (26.5%)**		
**DQA**	**n = 81**	**n = 89**		**n = 58**	**n = 31**		
**Homo**	61 (75.3%)	80 (89.9%)	**0.0142**	51 (87.9%)	29 (93.5%)	0.0822	0.4869
Hetero	20 (24.7%)	9 (10.1%)		7 (12.1%)	2 (6.5%)		
**DRB**	**n = 84**	**n = 91**		**n = 59**	**n = 32**		
Homo	62	53 (58.2%)	**0.0382**	34 (57.6%)	19 (59.4%)	**0.0484**	1.000
	(73.8%)					* *	* *
Hetero	22	38 (41.8%)		25 (42.4%)	13 (41.6%)		
	(26.2%)						

*WNND (West Nile Neuroinvasive Disease), WNF (West Nile Fever).

**Fisher's exact test.

## Discussion

In order to investigate the contribution of MHC alleles to the development of the infection with WNV, patients with symptoms of varying severity should be compared against asymptomatic WNV carriers. However, it became evident very early that gathering individuals that had been infected, yet not having sought medical advice and had been timely confirmed as asymptomatic, would not make a reliable control group. Therefore, we opted for the use of samples that were diagnosed negative for WNV infection and were collected from hospitals and health centers. Concerning our gene targeting, the difference of statistically significant associations with the disease, between *HLA-DQA1* (medium to low polymorphism) and *HLA-DRB1* (high polymorphism) confirmed the less-variable-selection we made. The combination of a small gene pool (WNV cases) with the high variability of the locus studied, incommodes the designation of specific alleles based on statistical rather than biological criteria.

The numbers of identified DPA, DQA, DRB alleles, in the 205 subjects studied were in agreement with the expected number of alleles in a given population, according to previous studies [27]. The majority of the alleles identified had been previously described. However, three alleles were novel in global scale. Due to the underrepresentation of Greek populations in MHC class II studies and in the IMGT/HLA Database [24] as well as the low sample size of the existing studies, ethnic-specific rare alleles could have been “missed out”. On the other hand, the existence of population-specific alleles suggests that differential selective pressures maintained these MHC allelic differences [28]. It has been shown that population-specific variation at the HLA class I loci is positively correlated with local pathogen richness [29]. Although, to our knowledge, the correlation between MHC class II genes and pathogen coevolution has not been studied in humans, this could be a valid case. Based on local MHC and parasite diversity it has been suggested that MHC differentiation between populations is, at least partly, a result of the exposure to different combinations of infectious agents [30].

The allelic frequencies in our cohort were compared with the equivalent frequencies in parts of the world (Europe, Western Asia, North Africa, Sub-Saharan Africa, and South Asia) that the Greek population mostly interacts with in the context of the prevailing migratory patterns (Table 1). Greece shares the same prevailing DPA allele (DPA1*01:03) with Europe. All alleles reported here follow Europe’s distribution. Only DPA1*01:05 was traced in our cohort at lower frequencies than in Europe.

Concerning the *HLA-DQA*1 locus, DQA1*01:02 and DQA1*01:01 prevailed in our cohort at higher frequencies than the ones in Europe. Interestingly, DQA1*01:02 and DQA1*01:01 reached frequencies previously recorded in Sub-Saharan Africa and Western Asia, respectively (Table 1).

The frequencies of nineteen DRB alleles fell within the ranges that have been so far recorded in Europe (Table 1). However, most of the frequencies observed matched the frequencies recorded in other populations as well; mostly with South and Western Asia. DRB1*11:04 exhibited the highest frequency of all DRB alleles in our cohort. This is in agreement with previous studies showing that DRB1*11:04 and DRB1*11:01 alleles are present in elevated frequencies in European Southeast populations compared with the other regions [27]. Greece, due to its geographic location, stands at the crossroads between Europe, Asia and Africa and at the convergence of different migratory routes. Since 2015, 1.012.560 refugees and immigrants have arrived in Greece [31]. This migration flow could shape molecular epidemics in the near future together with altered pathogen pressures driven by climate change.

In this context, it is not surprising that Greece harbors a number of novel alleles that counted for 8.3% of all the alleles identified in this study. All of them were low-frequency alleles, yet all of them exhibited a clear association with condition, being present solely in either controls or patients. Natural populations contain alleles in low frequency and several hypotheses have proposed that ‘balancing selection’ maintains such rare alleles in populations [32]. Amongst them, the hypothesis of negative frequency-dependent selection, which is postulating that individuals carrying rare alleles have a potential advantage, could explain the maintenance of the observed number of rare alleles [33].

The group of patients was more polymorphic than the control. The 36 MHC class II alleles identified in this study were unevenly distributed in controls and patients. 29 alleles were present in the control group something that comes in contrast with the 38 alleles in patient subjects. Accordingly, rare alleles (≤5%) compose 73.1% of the polymorphism found in patients and 46.2% in the control group. This observation contradicts the “rare-allele advantage” hypothesis which suggests that pathogens adapt to infect the most common host MHC alleles, leaving rare variants least infected [34]. Another possible explanation could be the recent and limited emergence of Flaviviruses in Greece. WNV appeared in Greek population in 2010. In 1927 the first case of Dengue virus was recorded in Greece. During a two-year epidemic >90% of the population developed symptoms and 1553 individuals died [35]. Usutu virus emerged in Europe after 2001. In Greece, no human cases were recorded [36]. No records for other zoonotic flaviviruses (Japanese encephalitis virus, yellow fever virus, Omsk hemorrhagic fever virus and Kyasanur forest disease virus) were found in Greece. The absence of incidence of flavivirus cases during a long period of time may not trigger any selection mechanisms establishing more favorable variants.

### WNV cases (WNND, WND) vs Control population

In humans, specific alleles of the *HLA-DPA*1, *HLA-DQA*1 and *HLA-DRB*1 loci have been associated with viral infections. Concerning infections caused by flaviviruses, DRB1*07, HLA-DR4 and DRB1*0901 seem to offer a protective advantage against dengue virus [18–20] while *HLA-DQ*1 is associated with dengue in Brazilian population [21]. Several of these alleles were identified in this study (DRB1*11:01, DRB1*11:04, DRB1*03:01, DRB1*15:02). Interestingly, *HLA-DQ*1 which is associated with dengue in the Brazilian population, corresponds to both DQA1*01:02 and DQA1*01:01, having a “susceptible” and “protective” character at the same time. This “bizzare” finding arose due to the discrepancy observed between our controls and the control populations retrieved from the database [24]. The control populations’ diversity renders the characterization of this allele relatively unreliable and compromises more generalized association with WNV infection.

Little information is available for the deceased patients. The high variability of the HLA-DRB1 locus, together with the small number of the analyzed cases made difficult to find association with WNV-induced mortality. DPA1*01:03 was the most common allele in all deceased, yet, the very high frequency of DPA1*01:03 (84.4%) across control and patient groups increases the probability of its exclusive identification in such a small cohort as the deceased.

Individuals heterozygous for HLA alleles may have a wider peptide binding repertoire and an increased capability to respond to more pathogen variants [27]. In accordance, the higher heterozygosity rate observed in a population would suggest lower infection rate and decreased disease severity. This is the case for *HLA-DQA1*. Almost 25% of control population was heterozygous while in WNV cases group heterozygosity was found in 10% of the individuals. The *HLA-DQA*1 heterozygosity may have a protective effect against WNV cases or infection. On the other hand, our results for *HLA-DRB1* come in contrast with the assumption that MHC heterozygotes have higher fitness compared with homozygotes; heterozygosity rate was 42% in patients unlike 25% that was found in controls. Surprisingly, DRB1 homozygosity seems to offer an advantage. A possible explanation is that not all homozygotes are at a disadvantage against an infectious disease. Although heterozygotes may have a wider peptide binding repertoire, certain alleles may offer stronger immune response against specific antigens [37].

The results of the present study provide strong indications that all three loci screened are implicated in the response to WNV infection. One DPA, one DQA and seven DRB alleles were exclusively identified in WNV cases, making them “susceptibility” candidates. In addition, one more DQA allele was present at significantly higher frequency in WNV cases, adding one more candidate to the “susceptibility” list. More importantly, “CNS-low-risk” and “CNS-high-risk” alleles, respectively, were identified between patients; DRB1*11:18, DRB11:02, DRB1*11:03 and DRB1*08:30 alleles were not detected in WNND patients, whereas DRB1*14:01, DRB1*16:02, DRB1*12:01, DBR1*13:03, DRB1*08 and DRB1*11:15 were detected solely in WNND patients. DQA1*01:02 also falls within the CNS-susceptible alleles as significantly more WNND patients bore it compared with WNF patients.

Using more than one control populations enhanced the associations observed and highlighted associations that were near border line of statistical significance. DQA1*01:01 was shown to be associated with both resistance and susceptibility to WNV infection in different cohort of Greek population. An explanation could be that different sub-populations have been influenced in different ways that could differentiate allelic frequencies, even in the same country. Another conjecture is that we cannot be sure about the Greek origin of our controls that could “unsettle” the frequency of alleles in our gene pool. Apart from this allele, DQA1*01:02 seems to offer a disadvantage concerning WNV infection while DQA1*05:01 plays a protective role against the infection. DRB1*14:01 was the only DRB allele with a protective role against WNV that was found in our study. However, several susceptibility alleles were verified such as DRB1*08:04, DRB1*14:04 and DRB1*16:01. This analysis further supports and amplifies the results found here.

This is the first study about genetic susceptibility to WNV infection in Greece and the study of more adaptive immunity genes may be necessary to draw the genetic pattern of susceptibility to infectious agents. Previous studies have underlined the role of several loci, besides MHC, that contribute to susceptibility status and/or disease severity. Some examples are *CCR5* (chemokine receptor type 5) [38], *OAS1* (2'-5'-oligoadenylate synthetase 1), *IRF3* (Interferon Regulatory Factor 3), *MX1* (MX dynamin-like GTPase 1) [39] and *KIR* [40].

Since 1999, West Nile encephalitis has rapidly spread in North America and Europe while Greece is considered a WNV-endemic country. So far, no human vaccine is available to control new WNV outbreaks and to avoid worldwide spreading of the virus. It has been shown that polymorphisms in the HLA genes restrict T lymphocyte responses to antigen thereby influencing vaccine virus-induced immunity [41]. Therefore, it is important to take into consideration the HLA variability in the development of a more effective vaccine-design. The study of the HLA identity of the populations at risk of infection could be an important piece of the puzzle of the re-emergence of mosquito-borne infections. In addition to the climate and weather change, the land use, the vectors abundance and the human behavior, genetic susceptibility may also help to estimate the severity of WNV re-emergence in a country.
